# Integrated shotgun sequencing and bioinformatics pipeline allows ultra-fast mitogenome recovery and confirms substantial gene rearrangements in Australian freshwater crayfishes

**DOI:** 10.1186/1471-2148-14-19

**Published:** 2014-02-03

**Authors:** Han Ming Gan, Mark B Schultz, Christopher M Austin

**Affiliations:** 1 School of Science, Monash University Malaysia, Jalan Lagoon Selatan, Bandar Sunway, 46150 Petaling Jaya, Selangor, Malaysia; 2 Faculty of Medical and Dental Health Sciences, The University of Melbourne, Bio21 Research Institute, 30 Flemington Rd, Parkville, Victoria 3010, Australia

**Keywords:** Freshwater crayfish, Mitochondrial genome characterization, Bench top sequencing, *Engaeus*, Parastacidae

## Abstract

**Background:**

Although it is possible to recover the complete mitogenome directly from shotgun sequencing data, currently reported methods and pipelines are still relatively time consuming and costly. Using a sample of the Australian freshwater crayfish *Engaeus lengana,* we demonstrate that it is possible to achieve three-day turnaround time (four hours hands-on time) from tissue sample to NCBI-ready submission file through the integration of MiSeq sequencing platform, Nextera sample preparation protocol, MITObim assembly algorithm and MITOS annotation pipeline.

**Results:**

The complete mitochondrial genome of the parastacid freshwater crayfish, *Engaeus lengana*, was recovered by modest shotgun sequencing (1.2 giga bases) using the Illumina MiSeq benchtop sequencing platform. Genome assembly using the MITObim mitogenome assembler recovered the mitochondrial genome as a single contig with a 97-fold mean coverage (min. = 17; max. = 138). The mitogenome consists of 15,934 base pairs and contains the typical 37 mitochondrial genes and a non-coding AT-rich region. The genome arrangement is similar to the only other published parastacid mitogenome from the Australian genus *Cherax*.

**Conclusions:**

We infer that the gene order arrangement found in *Cherax destructor* is common to Australian crayfish and may be a derived feature of the southern hemisphere family Parastacidae. Further, we report to our knowledge, the simplest and fastest protocol for the recovery and assembly of complete mitochondrial genomes using the MiSeq benchtop sequencer.

## Background

Sequencing of mitochondrial genomes has become an important endeavour for providing molecular resources for population genetic and phylogeographic studies [1-3]. With the rising number of sequenced mitogenomes there has also been increasing interest in using this information for phylogenetic studies [4-8]. Further, as more full mitochondrial genomes are sequenced, there are interesting patterns of mitochondrial gene order that demand explanation and are themselves an additional source of phylogenetic signal [7,8]. Until recent years, the recovery of whole mitochondrial genomes (mitogenomes) has been inefficient, with most approaches utilising either cloning or long range PCR (which may or may not be successful), followed by a series of Sanger sequencing [4,9-11]. More recently, however, the increasing power of Next Generation Sequencing (NGS) has allowed the amplification-free sequencing of whole mtDNA genomes [12,13]. This latter approach has been demonstrated using modest shotgun sequencing on platforms such the Illumina HiSeq, Illumina Genome Analyzer IIx (GA IIx) and Roche 454 [14-17]. In the case of Miller *et al*’s study, a mitogenome to 800 × coverage from 1/16^th^ of a plate was recovered using 454. Further, Berman *et al.* also showed that modest NGS outputs designed to identify microsatellite loci can be also be used to recover whole mitochondrial genomes [18]. Although these platforms represent a significant improvement over the conventional Sanger sequencing, the sample preparation and sequencing protocols are still relatively time consuming and laborious. Additionally, for the HiSeq platform, the run time can take up to 10 days. Coupled with its overly massive data output (600 gb), the HiSeq platform is not practical for the sequencing of mitogenomes. Although the data output is lower (90 gb) for the GA IIx platform, the operation of this system can represent a major technical challenge due to the lack of automation as compared to the HiSeq thus increasing the chance of human error and run failure. The sequencing chemistry of Roche 454 has been acknowledged to be susceptible to homopolymer issues hence representing a potential threat to the accuracy of the mitogenome assembly. Further, its high running cost and low data output also rendered it less cost-friendly for mitogenome sequencing. The development of the MiSeq benchtop sequencer and the timely introduction of MITObim [14], a low computationally demanding software for the assembly of mitochondrial genomes using a novel baiting and iterative mapping approach, serves as an impetus for the growth in NGS-based mitogenome assemblies. Although a successful mitogenome assembly using the MiSeq benchtop sequencer has been demonstrated recently, the library preparation steps for sequencing on the MiSeq were not covered in sufficient details nor was MITObim implemented in the assembly pipeline [19]. Using a sample of the Australian freshwater crayfish *Engaeus lengana*[20], we contribute to the growing interest in mtDNA genome sequencing by providing a detailed protocol for the fastest recovery, assembly and annotation of mitogenome using the MiSeq personal genome sequencer, MITObim software and MITOS annotation web service.

Australia has a diverse and distinctive freshwater crayfish fauna despite the continent’s aridity. One of the most intriguing genera of Australian crayfishes are the land yabbies from the genus *Engaeus*, which can complete their life cycle without access to surface water [20,21]. While the understanding of the evolution of *Engaeus* and other crayfishes have benefited from access to molecular data, only one full mitogenome is available for the southern hemisphere crayfish from the genus *Cherax*[4]. Major mitogenome rearrangements were identified in the species *Cherax destructor* compared to what is considered the Pan-crustacean plan [22]. A recent study indicates that northern hemisphere crayfish also have profound mitochondrial gene order rearrangements [22,23], albeit different to the rearrangements identified in *Cherax*. Kim *et al.* in their study of the marine lobster, *Homarus americanus,* emphasised the need for the sequencing of more mitogenomes from the superfamily Astacidae [22].

The purpose of this study is two-fold: first to demonstrate the simplest protocol, to our knowledge, for the recovery of whole mitochondrial genomes directly from shotgun sequencing reads using the MiSeq platform. This protocol requires only 50 ng of DNA extracted from a single ethanol-preserved specimen, without the need for mtDNA enrichment; and, secondly, we use the Australian freshwater crayfish *E. lengana* to investigate further mitochondrial genome evolution in parastacid freshwater crayfishes, building on the work of Miller *et al.*[4] who sequenced the *C. destructor* mitogenome.

## Results

### Mitogenome assembly, coverage and composition

**Figure 1:**
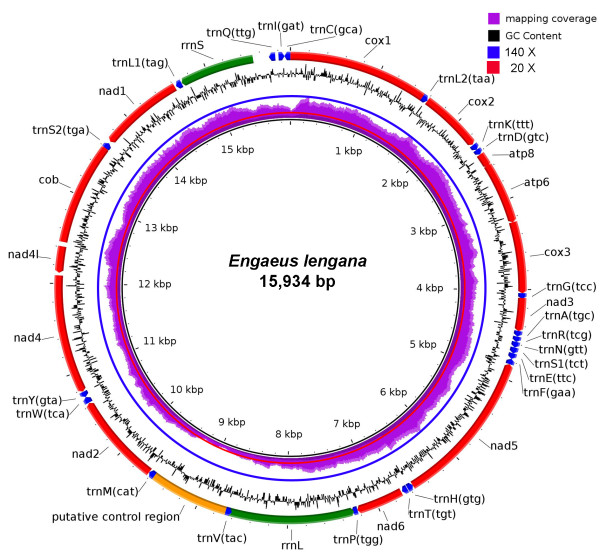
**The complete mitogenome of *E. lengana.*** BRIG visualization showing the mapping coverage, protein coding genes, rRNAs and tRNAs in the mitogenome of *E. lengana.* A lower mapping coverage can be observed at the putative control region. GC content is shown on the outer surface of the ring whereas AT content is shown on the inner surface.

A total of 4,761,100 paired-end reads amounting to approximately 1.2 giga bases of raw sequence data were generated from an *E. lengana* library*.* The MITObim selective-assembly of the raw reads resulted in the recovery of the complete mitochondrial genome of *E. lengana* [GenBank:KF546209] consisting of 15,934 bp (AT content of 66.27%). We also undertook a *de novo* assembly followed by BLAST against a fragment of the 16S rRNA gene region, which led to the recovery of the same mitogenome sequences but at the cost of significantly greater computational resources. The mitogenome contains 13 protein coding, two ribosomal RNA and 22 tRNA genes (Table 1 and Figure 1). Based on Bowtie2 mapping, a total of 6,442 reads (0.1% of total reads) were mapped to the constructed mitogenome giving relatively evenly distributed read coverage. The major exception is the putative control region spanning from position 8,686 to 9,556, where the coverage range is from 17 to 50 (Figure 1). The overall AT content of the *E. lengana* α-strand and β-strand is 62.92% and 66.99% respectively. A total of 1,264 non-coding nucleotides were observed over multiple intergenic regions. The longest non-coding region (976 bp) was located between the tRNA^met^ and tRNA^val^ genes. Given that the AT content in this non-coding region is significantly higher than the rest of the mitogenome (Chi-square test, P < 0.001), it most likely represents the control region and is located in a similar region to the control region identified in *C. destructor*.

**Table 1:** **The mitochondrial genome organization of *E. lengana***

**Gene**	**Start**	**End**	**Orientation**	**No. of nt (bp)**	**Intergenic sequence**	**Putative initiation/termination codon**
cox1	1	1566	Forward	1566	−31	Undetermined/TAA
trnL2(taa)	1535	1601	Forward	67	2	
cox2	1603	2292	Forward	690	8	ATG/TAA
trnK(ttt)	2300	2364	Forward	65	1	
trnD(gtc)	2365	2427	Forward	63	2	
atp8	2429	2587	Forward	159	−12	ATG/TAA
atp6	2575	3255	Forward	681	0	TTG/TAA
cox3	3255	4043	Forward	789	−2	ATG/TAA
trnG(tcc)	4041	4104	Forward	64	−10	
nad3	4094	4459	Forward	366	−2	ATA/TAG
trnA(tgc)	4457	4518	Forward	62	2	
trnR(tcg)	4520	4579	Forward	60	0	
trnN(gtt)	4579	4642	Forward	64	1	
trnS1(tct)	4643	4708	Forward	66	0	
trnE(ttc)	4708	4771	Forward	64	0	
trnF(gaa)	4771	4832	Reverse	62	1	
nad5	4833	6560	Reverse	1728	0	ATG/TAA
trnH(gtg)	6560	6625	Reverse	66	8	
trnT(tgt)	6633	6694	Forward	62	13	
nad6	6707	7225	Forward	519	0	ATC/TAA
trnP(tgg)	7225	7291	Forward	67	−25	
rrnL	7266	8646	Reverse	1381	−30	
trnV(tac)	8616	8685	Reverse	70	872	
trnM(cat)	9557	9626	Forward	70	2	
nad2	9628	10635	Forward	1008	5	ATG/TAA
trnW(tca)	10640	10709	Forward	70	3	
trnY(gta)	10712	10773	Reverse	62	5	
nad4	10778	12088	Reverse	1311	30	ATA/TAA
nad4l	12118	12408	Reverse	291	51	ATA/T*
cob	12459	13610	Forward	1152	−16	ATG/TAA
trnS2(tga)	13594	13658	Forward	65	23	
nad1	13681	14610	Reverse	930	17	ATG/TAA
trnL1(tag)	14627	14693	Reverse	67	4	
rrnS	14697	15522	Reverse	826	176	
trnQ(ttg)	15698	15767	Reverse	70	37	
trnI(gat)	15804	15868	Forward	65	0	
trnC(gca)	15868	15933	Reverse	66	1	

### Proteins and codons

The typical 13 mitochondrial protein coding genes (11,190 bp) were identified spanning 70.22% of the mitogenome. The predominant stop codon is TAA with the exception being for *nad2* and *nad4l,* which use TAG and an incomplete termination codon respectively. Incomplete termination codons are fairly common in metazoan mitogenomes and can be converted into a potential stop codon via polyadenylation to TAA [24] (Table 1). In general, the composition of AT bases is higher in the 3^rd^ codon (Chi-square test, p < 0.001). The composition of guanine base at the 3^rd^ codon on the beta-strand is substantially higher than that from the alpha strand (21.42% vs 3.81%) and the reverse for the composition of the cytosine base (2.75% vs 25.80%) (Chi-square test, p < 0.001) (Table 2).

**Table 2:** **Base composition of protein coding genes in the *E. lengana *mitogenome**

	**A**	**C**	**G**	**T**	**(A + T)**	**(G + C)**
All genes						
1st codon	27.59	18.53	22.47	31.42	59.01	41.00
2nd codon	17.86	21.15	16.92	44.08	61.94	38.07
3rd codon	32.9	17.02	10.51	39.57	72.47	27.53
Total	26.11	18.9	16.63	38.36	64.47	35.53
Genes encoded on α-strand						
1st codon	28.4	23.12	19.87	28.61	57.01	42.99
2nd codon	18.61	24.59	14.07	42.73	61.34	38.66
3rd codon	33.59	25.8	3.81	36.8	70.39	29.61
Total	26.87	24.5	12.58	36.05	62.92	37.08
Genes encoded on β-strand						
1st codon	26.27	11.06	26.69	35.99	62.26	37.75
2nd codon	16.62	15.56	21.55	46.27	62.89	37.11
3rd codon	31.76	2.75	21.41	44.08	75.84	24.16
Total	24.88	9.79	23.22	42.11	66.99	33.01

### Gene order

**Figure 2:**
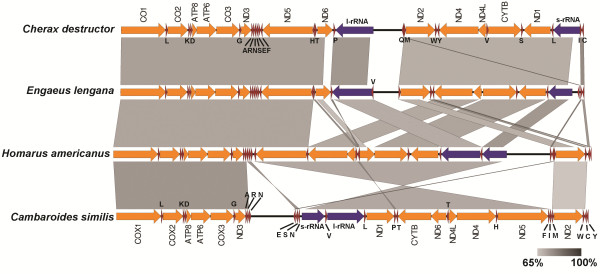
**The gene organization in the *E. lengana *mitogenome and its high similarity to *C. destructor*.** Linear genome comparison of the mitogenomes of *E. lengana*, *C. destructor* (Accession number: AY383557), *C. similis* (Accession number: JN991196) and *Homarus americanus* (Accession number: NC_015607) and. Regions of similarity based on BLASTn which satisfy the E-value threshold of less than 0.001 are shown.

The gene organization in the *E. lengana* mitogenome is almost identical to that of *C. destructor,* the only other parastacid crayfish, for which a whole mitogenome is available with the exception being the positions of tRNA^met^ and tRNA^val^. In *E. lengana*, the tRNA^met^ is located upstream of *nad2* while in *C. destructor,* it is located downstream of the ssuRNA. The tRNA^val^ gene is located downstream of the lsuRNA of *E. lengana* but in *C. destructor*, it is located upstream of the *cob* gene (Figure 2). It should be noted that the position of tRNA^val^ was only putatively identified by Miller *et al.*[4]. Notably, in both *C. destructor* and *E. lengana*, the ribosomal RNA genes are separated by *nad2, nad4, nad4l, cob* and *nad1* genes, which is not observed in the mitogenome of other members of the infraorder Astacidea such as the American lobster, *Homarus americanus* or the northern hemisphere crayfish, *Cambaroides similis* and *Procambarus clarkii*[22,23].

### Non-coding RNA

**Figure 3:**
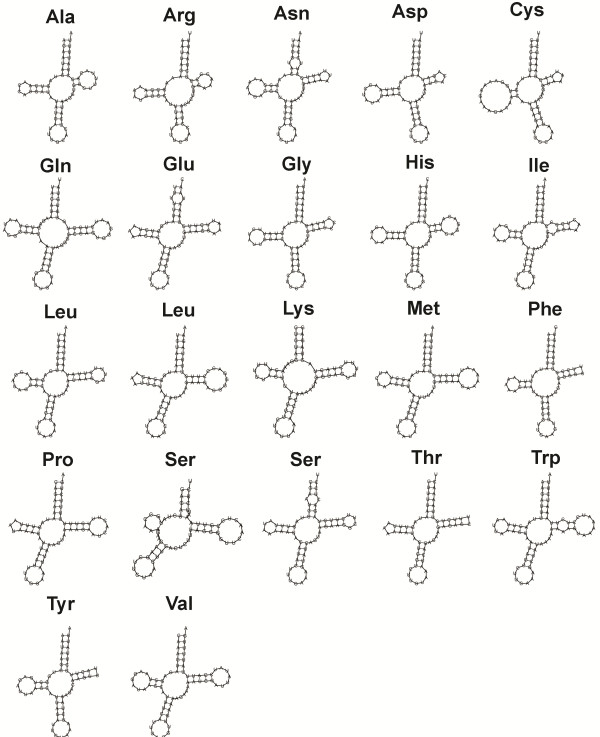
**Predicted tRNA structures.** 22 tRNAs are identified in the mitogenome of *E. lengana* and their cloverleaf secondary structures of tRNAs are inferred with Infernal software module, which is part of the MITOS annotation pipeline.

A total of 22 tRNA genes were predicted in the mitogenome of *E. lengana* with length ranging from 60 to 70 bp. Inferred cloverleaf secondary structures of tRNAs are presented in Figure 3. In one of the tRNA^ser^ structures, only a limited trace of the “DHU” arm is present. The large ribosomal rRNA (1,381 bp) is flanked by tRNA^pro^ and tRNA^val^ while the small ribosomal (826 bp) is flanked by tRNA^leu^ and tRNA^met^.

## Discussion

The time taken from sample extraction to submission-ready full mitogenome sequence was only three days, and hands-on time was less than four hours. This makes our workflow one of the most efficient thus far reported [11,16,19,22,25]. The low-input DNA-quantity requirement and the simplicity of this protocol are particularly advantageous for new users as it minimizes the risk of failure and reduces sample quantity requirements. The MiSeq data output per run (4.5-7.5 gb) will enable sequencing of multiple samples via barcoding. Given that the data generated in this study (97-fold coverage) is more than twice that required to confidently deduce mitogenomes using MiSeq [18] , this means $100 mt genomes are now obtainable. According to the animal genome database (http://www.genomesize.com), the average genome size of a crayfish can range from 3.5 to 6 gb. Therefore, the percentage of reads mapping to the mitochondrial genome (0.1%) in *E. lengana* can be considered to be on the high side for enrichment-free shotgun sequencing. This may also reflect the use of muscle tissue, which has a high proportion of mitochondria. With additional pre-sequencing sample processing including mitochondrial enrichment [25], this would further reduce per-sample sequencing cost by allowing even more samples to be processed per run. Using this pipeline, we were also able to recover complete mitogenomes from very different organisms including the Australian Macquarie Perch, *Macquaria australasica* [EMBL:HG799088] and the Viet Nam Snout Otter Clam, *Lutraria rhynchaena* [EMBL:HG799089]. If further studies demonstrate wide utility of our methodology across a broad range of animal taxa, there exists the possibility of up-scaling DNA barcoding [26] from a small fragment of the mitochondrial COI gene to the level of the entire mitogenome.

For situations where there is no close relative to provide a “bait” sequence for MITObim, we demonstrated that a *de novo* assembly followed by BLAST search against the conserved mitochondrial genes (such as 16S rRNA) was effective for identifying mitogenome fragment(s) from the generated sequences. These can then in turn be used as the bait for a MITObim assembly.

This study brings the number of freshwater crayfish mitogenomes to five, comprising three from the northern hemisphere superfamily the Astacoidea (*Procambarus* and *Cambaroides*) [23] and now two from the southern hemisphere superfamily Parastacidae (*Engaeus* and *Cherax*) [4]. The representatives of each superfamily have substantial gene order rearrangements in comparison to each other and their nearest relative from the marine clawed lobsters, *Homarus americanus* (Superfamily: Nephropoidea). The latter has what is deemed to be the more primitive pan-crustacean gene order [22]. It is now apparent that Australian, and perhaps parastacid crayfish more generally, have one of the most elaborate gene order rearrangements so far discovered in the Arthropoda. It is possible that the translocation and inversion of the RNA genes is a distinctive feature of Australian crayfish. Future studies of freshwater crayfishes will likely benefit from the phylogenetic signal provided in mitochondrial gene order rearrangements [11,27,28].

## Conclusion

In conclusion, this study demonstrates that benchtop sequencers can be used to obtain fast and relatively inexpensive generation of mt DNA sequences using shotgun sequencing without mitochondrial enrichment. We also show that MITObim [14] is effective at recovering mitogenomes from raw benchtop sequencer output. Previously reported significant gene order rearrangements in Australian crayfish are confirmed. The further sequencing of mitogenomes of southern hemisphere crayfish (Parastacidae) and additional representatives of the northern hemisphere families (Cambaridae and Astacidae) will undoubtedly contribute to our phylogenetic knowledge of this significant group of crustaceans. Lastly, we predict that improvements to our workflow and increased output from benchtop sequencers will further reduce the cost of reconstructing mitogenomes to much less than $100 per mitogenome.

## Methods

### Genomics DNA extraction

Approximately 40 mg of tail muscle tissue was dissected from an ethanol-preserved specimen of *E. lengana* collected from northern Tasmania (−41.00877 ° S, 144.66869 ° E). Total genomic DNA was extracted using DNAeasy Blood and Tissue Kit (Qiagen, Germany) following the manufacturer’s instructions with minor modification. EB rather than AE buffer was used to avoid possible interference of EDTA with the Nextera enzyme.

### Molecular procedures and sequencing

The purified genomic DNA was quantified with Qubit HS (Invitrogen, USA) and normalized to 2 ng/μL. The normalized DNA was processed using Nextera-based library preparation (Illumina, USA) following the manufacturer’s instructions. Quantification and size estimation of the library was performed on a Bioanalyzer 2100 High Sensitivity DNA chip (Agilent, USA). Next, the library was normalized to 2 nM and sequenced on the MiSeq Benchtop Sequencer (2 × 250 bp paired-end reads) (Illumina, USA).

### Mitogenome assembly and annotation

**Figure 4:**
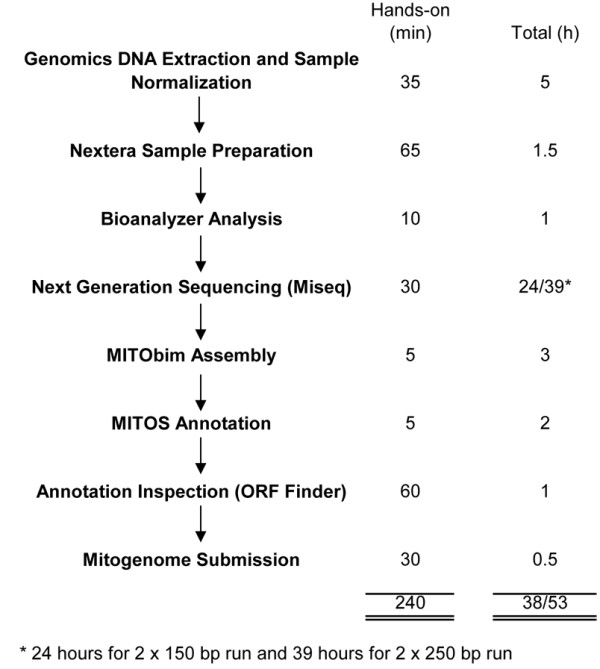
**Mitogenome recovery workflow.** Steps involved, hands-on and total time required from raw tissue sample to annotated mitogenome.

The mitochondrial genome was reconstructed with MITObim [14] using the COI gene sequences of *Engaeus sericatus* (GenBank Accession number: FJ965960) as the seed reference and using the parameters: “--trim”, “–pair” and “--noshow”. As an alternative approach to using a seed reference we completed a de novo assembly using CLC Bio (CLC Genomics Workbench 5, Denmark) and searched for matches to the conserved 16S rRNA gene. The assembled mitogenome was then manually inspected for repeats at the beginning and end of the assembly to infer circularity. The mitogenome was annotated with MITOS [29] followed by manual validation of the coding regions using the NCBI ORF Finder (http://www.ncbi.nlm.nih.gov/projects/gorf/). Based on ORF Finder result, the sqn file generated from MITOS was edited and submitted to NCBI. The steps involved, hands-on time and total time required from tissue sample to annotated mitogenome ready for NCBI submission is illustrated in Figure 4.

### Mitogenome visualization and linear comparison

The circular mitogenome of *E. lengana* was visualized with Blast Ring Image Generator (BRIG) [30]. Mapping statistic was generated by mapping the raw reads against the assembled mitogenome using Bowtie2 [31]. SAM file output from Bowtie2 was subsequently used to visualize the mapping coverage via BRIG [30]. Linear mitogenome comparison of *E. lengana, C. destructor, Cambroides similes* and *H. americanus* (Figure 2)*,* was performed using EasyFig2.1 (BLASTn, default setting) [32].

## Competing interests

H.M.G is an ex-employee of ScienceVision Sdn Bhd. ScienceVision Sdn Bhd is the sole distributor of Illumina products in Malaysia. The other authors declare no competing interests.

## Authors’ contributions

HMG and CMA conceived and designed the experiments. HMG performed the sequencing and analyzed the data. MBS and CMA contributed reagents, materials and analysis tools. All authors discussed the results and wrote the manuscript. All authors read and approved the final manuscript.
